# Cell Wall Heterogeneity in Root Development of *Arabidopsis*

**DOI:** 10.3389/fpls.2016.01242

**Published:** 2016-08-17

**Authors:** Marc Somssich, Ghazanfar Abbas Khan, Staffan Persson

**Affiliations:** ^1^School of Biosciences, University of MelbourneMelbourne, VIC, Australia; ^2^Department of Plant Molecular Biology, University of LausanneLausanne, Switzerland

**Keywords:** cell wall, root, development, meristematic zone, elongation zone, differentiation zone, nutrients

## Abstract

Plant cell walls provide stability and protection to plant cells. During growth and development the composition of cell walls changes, but provides enough strength to withstand the turgor of the cells. Hence, cell walls are highly flexible and diverse in nature. These characteristics are important during root growth, as plant roots consist of radial patterns of cells that have diverse functions and that are at different developmental stages along the growth axis. Young stem cell daughters undergo a series of rapid cell divisions, during which new cell walls are formed that are highly dynamic, and that support rapid anisotropic cell expansion. Once the cells have differentiated, the walls of specific cell types need to comply with and support different cell functions. For example, a newly formed root hair needs to be able to break through the surrounding soil, while endodermal cells modify their walls at distinct positions to form Casparian strips between them. Hence, the cell walls are modified and rebuilt while cells transit through different developmental stages. In addition, the cell walls of roots readjust to their environment to support growth and to maximize nutrient uptake. Many of these modifications are likely driven by different developmental and stress signaling pathways. However, our understanding of how such pathways affect cell wall modifications and what enzymes are involved remain largely unknown. In this review we aim to compile data linking cell wall content and re-modeling to developmental stages of root cells, and dissect how root cell walls respond to certain environmental changes.

## Introduction

### Plant Roots

Plant roots of most plant species share a similar basic architecture. They are organized in a fixed radial pattern of different cell types that persist throughout the root and that already is established during embryogenesis (Huang and van Steveninck, 1990; Dolan et al., 1993; Feldman, 1994; Morita and Nemoto, 1995). In brief, the vascular bundle cells of the stele make up the center of plant roots. These cells are surrounded by the endodermis; a single cell layer that forms a protective barrier for the vascular bundle. The endodermis is in turn encased by cortex cells, which may consists of a single cell layer as in *Arabidopsis*, but that also can be several cell layers thick as in maize or barley. The outermost cell layer is the epidermis, which functions as a selective barrier between the root and its environment (Huang and van Steveninck, 1990; Dolan et al., 1993; Feldman, 1994; Morita and Nemoto, 1995).

**FIGURE 1:**
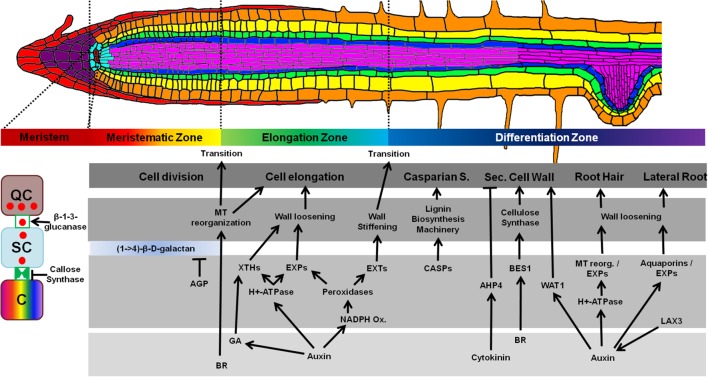
***Arabidopsis* root architecture and processes that influence cell wall deposition**. Upper panel; different developmental zones of the root are shown from the meristem (dark red) to DZ (dark blue). Lower panel: (dark gray box), characteristics of each zone are indicated (cell division, cell elongation, etc.), followed by associated cell wall modifications (lighter gray box) and proteins, and the underpinning hormone signaling pathways (lowest, light gray box). Far left panel, a QC-derived signal (red circles) might move to neighboring stem cells (SCs), possibly through PD to maintain stem cell fate, but is blocked from further travel to the stem cell daughter cell (C) by PD exclusion. AGP, arabinogalactan-protein; XTHs, xyloglucan endotransglycosylases/hydrolases; EXP, expansins; EXT, extensins; CASPs, CASPARIAN STRIP DOMAIN PROTEINS; AHP4, ARABIDOPSIS HISTIDINE-CONTAINING PHOSPHOTRANSFER FACTOR 4; BES1, BRI1-EMS-SUPPRESSOR1; WAT1, WALLS ARE THIN1; BRs, brassinosteroids; GAs, gibberellins.

Roots also display a well-defined developmental gradient along their longitudinal axes, with young cells being close to the root tip and the older cells at the root base (**Figure 1**; Schiefelbein and Benfey, 1991). The RAM, located close to the root tip in the MZ is the stem cell niche of the roots. Here, a single layer of stem cells is maintained by four central cells, the QC (Clowes, 1954). Only cells in direct contact with the QC remain in a stem cell state, possibly due to signals stemming from the QC (van den Berg et al., 1997). The stem cells distal to the QC form, and continuously renew, the protective root cap, while the stem cells proximal to the QC produce the main root body (Dolan et al., 1993). New cells that are displaced from the QC will no longer be maintained in a stem cell state and enter the TZ, where the cells divide rapidly and produce more cells. Once they have passed the TZ, the cells will cease to divide and enter the EZ. Here, cells elongate until they reach their final size and obtain specific functions in the DZ. In the DZ, some cells of the pericycle form lateral roots, epidermal cells may form root hairs, and the CS will develop between cells of the endodermis (Schiefelbein and Benfey, 1991).

### The Plant Cell Wall

**FIGURE 2:**
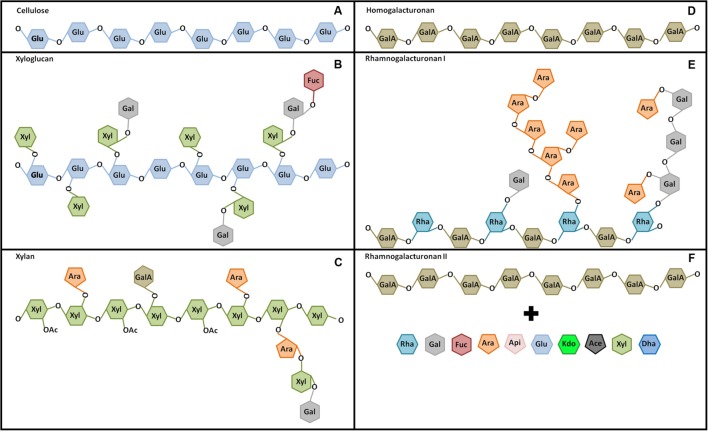
**The major cell wall polymers. (A)** Cellulose consists of long chains of β-(1→4)-linked D-glucose (Glu). **(B)** The hemicellulose xyloglucan consists of a Glu backbone (β-(1→4)-linked) with Glu-(6→1)-xylose (Xyl), Glu-(6→1)-Xyl-(2→1)-galactose (Gal) or Glu-(6→1)-Xyl-(2→1)-Gal-(2→1)-fucose (Fuc) side-chains. **(C)** The hemicellulose xylan consists of a β-(1→4)-linked Xyl backbone with arabinose (Ara), galacturonic acid (GalA) or Ara-(2→1)-Xyl-(2→1)-Gal chains linked to the carbon in position 2 or 3. Additionally, some Xyl are *o*-acetylated (OAc). **(D)** The pectin HG consists of α-(1→4)-linked GalA polymers. **(E)** The pectin RGI consists of a backbone of GalA-(4→1)-Rhamnose-(2→1)-GalA repeats with Rha-(4→1)-Gal or Rha-(4→1)-Ara side-chains of variable length. **(F)** The pectin RGII consists of a HG backbone with a wide variety of side-chains containing Rha, Gal, Fuc, Ara, apiose (Api), Glu, 3-deoxy-D-manno-oct-2-ulosonic acid (Kdo), *O*-acetyl-L-aceric acid (Ace), Xyl and 3-deoxy-D-lyxo-heptulosaric acid (Dha).

Every plant cell is encased by cell walls, which provide structural support, e.g., preventing cells from bursting due to internal turgor, enabling roots to push through the soil, and protecting cells against the environment (Ivakov and Persson, 2012). Plant cell walls are mostly made up of three classes of polysaccharides: cellulose, hemicelluloses and pectins. Cellulose consists of para-crystalline microfibrils made of β-(1→4)-linked D-glucose (**Figure 2A**) that are synthesized at the plasma membrane by CesA complexes (McFarlane et al., 2014). The microfibrils serve as the scaffold that maintain cell wall strength and are cross-linked by matrix polysaccharides (Ivakov and Persson, 2012). More specifically, recent work suggests that hemicelluloses, such as xyloglucans, may tether the microfibrils at distinct junctions (Park and Cosgrove, 2015). The major hemicelluloses in primary cell walls are xyloglucans, xylans, mixed-linked glucans and mannans, depending on species, and tissue and cell type studied (**Figures 2B,C**; Scheller and Ulvskov, 2010). The backbones of these polymers are typically β-(1→4)-linked sugars, making them similar to the cellulose strands. These polysaccharides are synthesized in the Golgi apparatus and then secreted to the apoplast, where they become incorporated into the wall (Scheller and Ulvskov, 2010). Finally, pectins form a dense aqueous wall matrix and connect cell wall polymers around and between cells. Pectins are typically sorted into three classes: HGs, RGI, and RGII (**Figures 2D–F**; Mohnen, 2008). Pectins are preferentially built around α-(1→4)-linked D-galacturonic acid backbones that can be diversely substituted. HG consists of linear chains of α-(1→4)-linked D-galacturonic acid, which can be methyl- or acetyl-esterified (**Figure 2D**). RGI consist of α-(1→4)-linked D-galacturonic acid-α-rhamnose-(1→2)-linked repeats with galactose and arabinose sidechains (**Figure 2E**), while RGII can form highly complex and diverse polymers, including a plethora of sugars and sidechains, with α-(1→4)-linked D-galacturonic acids serving as the central structure (**Figure 2F**; Atmodjo et al., 2013). Like hemicelluloses, pectins are synthesized in the Golgi apparatus, from where they are transported to the cell wall (Mohnen, 2008).

Primary cell walls are produced during cell growth and division at the growing cell plate, and are thin and elastic (Cosgrove, 1997). Following differentiation, some primary walls are enforced by the production of secondary cell walls (Li et al., 2016). These secondary walls are much thicker than primary walls and rigid in nature, thereby providing strength and stability to the cell and the plant. Most sclerenchymatous cells are encased in secondary walls, with the prime example being xylem vessel cells in the plant vasculature. Here, the secondary walls allow for water to be transported through the interconnected vessel elements and thus mediates highly efficient water transport through the plant (Ivakov and Persson, 2012). Structurally, secondary walls are different from primary walls in that they mainly contain cellulose, xylans and the polyphenolic structure lignin, which is made from a network of coniferyl-, sinapyl-, and/or *p*-coumaryl alcohols (Kumar et al., 2015). Lignin provides extra strength to the walls and makes them waterproof.

While the principal building blocks for the cell walls are similar, functional needs of different cells lead to a diverse set of cell wall polymers being produced around individual cells. It can therefore be assumed that the cell walls are heterogenous when one compares them across different cell types and developmental stages. Indeed, the polysaccharide content changes throughout the developmental zones of roots, and across specialized cell types (Freshour et al., 1996). However, how these differences come about and what is triggering them remains largely unknown. Furthermore, a major function of roots is to absorb water and nutrients from the soil and the nutrient availability affects root growth. Hence, nutrient availability must therefore also impact cell wall plasticity and its content. Nevertheless, a firm understanding for how nutrient availability influences the cell wall is lacking. In this review, we aim to compile and synthesize developmental and environmental pathways that affect cell wall content in different cell types of the main body of *Arabidopsis* roots. Due to many recent reviews on root hair growth and development (Ishida et al., 2008; Mendrinna and Persson, 2015; Mangano et al., 2016; Salazar-Henao and Schmidt, 2016), and on hormonal effects on root growth (Sánchez-Rodríguez et al., 2010; Ubeda-Tomás et al., 2012; Lavenus et al., 2013; Tian et al., 2014; Pacifici et al., 2015), we have largely excluded these fields from this review.

## The Root Meristematic and Transition Zones

The *Arabidopsis* RAM holds four central QC cells, which maintain a single surrounding stem cell layer (termed ‘initials’; Clowes, 1954). While the QC cells are mitotically less active, the initials continuously divide to produce new cells, which enter the pathway toward differentiation (van den Berg et al., 1997). Given these two opposing traits, it would be expected that the cell walls surrounding the QC and initial cells are very different from each other. Curiously, there is very limited information on the composition of the cell walls surrounding the QC and the initials. It has been suggested that a stem cell signal is generated in the QC and that this signal is transmitted via PD to the directly adjacent cell file to keep these cells in a stem cell state (Barclay et al., 1982; van den Berg et al., 1997; Stahl et al., 2013). The transmission of such signal would most likely depend on the opening or closing ability of the PDs, which is regulated by callose content, a β-1-3-glucan, in the cell walls surrounding the PDs (Wolf et al., 1991).

Apart from the QC and initials, the MZ also comprises several layers of newly formed cells that have begun to differentiate and reached the TZ (Schiefelbein and Benfey, 1991). These cells have high mitotic activity, and are undergoing several rounds of cell division. Here, the cells are surrounded by a thin primary cell wall, which allows for frequent divisions (Baluška et al., 1996). Plant cells determine the position of the new wall via the phragmoplast, a microtubule-based structure that is formed between the two daughter cells and serves as a scaffold for the assembly of new membranes and a new wall referred to as the cell plate (Frey-Wyssling et al., 1964; Müller et al., 2009). Cell plate material, as well as enzymes that synthesize new wall material at the cell plate, are either secreted via Golgi-derived vesicles, or via endocytic vesicles harboring recycled cell wall material, along the phragmoplast. During the early stages of wall assembly, callose is the predominant polysaccharide (Miart et al., 2014; Drakakaki, 2015). Most likely it is synthesized directly at the site of the forming cell plate and serves to stabilize the wall in its earliest stages. Later, the cell plate is consolidated by a cellulose/hemicellulose network, and finally fuses with the parental cell wall, concomitantly with callose degradation (Drakakaki, 2015). The pectins, and to some extent hemicelluloses, form the middle lamella, which support intercellular junctions. These structures may already be detected at very early cell plate stages, and further accumulate until the cross wall is fully established (Drakakaki, 2015). Cellulose is similar to callose synthesized directly at the cell plate by CesA complexes, which are secreted or recycled via *trans*-Golgi network derived vesicles (Miart et al., 2014). While cellulose is largely absent from very young cell plates, it gradually replaces callose, until it eventually becomes the major load-bearing structure of the cross walls (Drakakaki, 2015).

Cell walls in the TZ are marked by the occurence of pectic (1→4)-β-D-galactan (McCartney et al., 2003). This appears to be somewhat specific for the TZ, where the cells are reprogrammed from primarily mitotic activity to elongation. As a marker for the transition to cell expansion, deposition of this cell wall epitope is correlated with root growth, as mutants impaired in root growth contain reduced (1→4)-β-D-galactan in their cell walls, and since hormone mediated growth inhibition resulted in reduced (1→4)-β-D-galactan levels (McCartney et al., 2003). However, treatment with the AGP-binding β-glucosyl Yariv reagent, which blocks AGP dependent cell elongation, resulted in an overall increase and persistence of (1→4)-β-D-galactan epitopes in the walls of also the EZ and DZ, indicating that (1→4)-β-D-galactan removal from the wall is blocked by this treatment, and that (1→4)-β-D-galactan may also result in failure of the cells to elongate (McCartney et al., 2003).

In the interphase between TZ and EZ, cells are becoming primed for rapid cell growth by re-organization of the microtubule array: cortical microtubules are typically haphazardly oriented in MZ and TZ cells. However, when the cells exit the TZ the microtubules become largely transversely oriented to the growth axis (Verbelen et al., 2006). As cortical microtubules guide cell wall synthesis this reorientation is important to support directed cell growth (Landrein et al., 2013). In particular, the microtubules guide the deposition of cellulose, the major load-bearing cell wall component, via the protein CSI1/POM2 (Hauser et al., 1995; Bringmann et al., 2012). Hence, the transversely organized microtubule array will restrict radial cell expansion by directing the deposition of cellulose microfibrils (Bringmann et al., 2012). One protein that might be involved in connecting microtubule and cellulose microfibril orientation is COB (Roudier et al., 2005). COB was originally identified in a genetic screen because of its short root phenotype, as *cob* mutants are defective in cell elongation (Benfey et al., 1993). The reduced cell elongation correlated with a disorganization of cellulose microfibril alignment, resulting in partly isotropic cell expansion (Roudier et al., 2005). Accordingly, mutants that affect microtubule organization and cellulose synthesis display severe defects in anisotropic root growth, including for example *bul* (Catterou et al., 2001) and *bot1* mutants (Bichet et al., 2001). In *bot1* root cells, the microtubules do not align in transverse arrays at the end of the MZ but remain largely random, which results in impaired anisotropic cell growth (Bichet et al., 2001). Furthermore, the cellulose content was reduced in *bot1* mutant plants, a feature it shares with the *prc1* mutant (Fagard et al., 2000). *prc1* mutants develop shorter roots with swollen cells in the EZ, hinting toward a defect in cell elongation (Desnos et al., 1996). *PRC1* has been shown to encode CesA subunit 6 and accordingly, the short-root phenotype is accompanied by reduced cellulose content (Fagard et al., 2000). Interestingly, *prc1* mutants were also defective in cortical microtubule organization in the root, indicating that while CesA movement is guided by microtubules, the activity of the CesAs might provide feedback on microtubule organization (Paredez et al., 2008). Microtubule organization is, furthermore, important for the movement of the SHR transcription factor from the stele to the endodermis (Wu and Gallagher, 2013). *shr* mutant plants fail to specify the endodermal cell layer, and are unable to maintain meristematic activity, and therefore only produce short roots with a reduced MZ and EZ (Benfey et al., 1993; Helariutta et al., 2000).

## The Root Elongation Zone

In the EZ, cells rapidly expand along the longitudinal axis via the cellulosic framework established in TZ and maintained during the EZ (Schiefelbein and Benfey, 1991). While this framework prevent lateral expansion of the cells it needs additional remodeling to allow elongation (Ledbetter and Porter, 1963). This is mainly achieved by cell wall enzymes, which remodel and loosen the cell wall and thus allow for cell wall extension during increase in turgor pressure (Ledbetter and Porter, 1963; Cosgrove, 2005). The phytohormone auxin is involved in the regulation of these processes by promoting acidification of the cell wall, according to the so called acid growth hypothesis. Here, proton-pumps are activated by auxin and the subsequent acidification of the wall triggers the activity of EXPANSINs (Hager et al., 1971, 1991; Shcherban et al., 1995; Park and Cosgrove, 2012). While there is only very limited information about this process in *Arabidopsis* roots, EXPANSINSs loosen the cell wall at cellulose-xyloglucan junctions, allowing for slippage of the cellulose microfibrils and thus to cell wall extensibility (McQueen-Mason et al., 1992; Shcherban et al., 1995; Park and Cosgrove, 2015).

Apart from EXPANSINs, XTH family proteins can aid in loosening of cell walls. The members of this family can be subdivided according to their enzymatic activity: while some members exclusively possess endohydrolase activity to irreversibly shorten xyloglucan chains (referred to as XEHs), most possess endotransglucosylase activity to “break and re-ligate” xyloglucan polymers (referred to as XETs; Rose et al., 2002; Eklöf and Brumer, 2010). Strong XET activity, especially in the root EZ, has indeed been shown *in vivo* by monitoring the incorporation of sulforhodamine-labeled xyloglucans into the growing cell wall of *Arabidopsis* roots (Vissenberg et al., 2000, 2003). In contrast to the EXPANSINs, activity of XETs appears to be largely maintained via transcriptional regulation (Vissenberg et al., 2005). Several hormones, including auxin, BRs, ABA, GA and ethylene, affect expression of different *XTH* genes. Of the 33 XTH members in *Arabidopsis*, *XTH17-20* seem to be the major contributors to root growth and for normal root development (Vissenberg et al., 2005). Of these, *XTH17* and *18* are expressed specifically in the EZ and DZ, while *XTH19* is expressed throughout the root and *XTH20* mainly in the DZ (Vissenberg et al., 2005). Expression of *XTH17, 18*, and *19* is induced by auxin and BR treatment, and *XTH18*, *XTH14*, and *XTH31* by GA (Yokoyama and Nishitani, 2001; Vissenberg et al., 2005), suggesting that XET activity might be related to hormone signaling. Consistent with this, expression of a gain-of-function version of the repressor of auxin signaling, *iaa17*, resulted in repression of *XTH17* and *XTH20* expression and reduced root growth (Overvoorde et al., 2005). No XTH18 knockout lines have been reported, perhaps indicating an important role of XTH18 function during plant, and possibly root, development. However, *XTH18* RNAi lines, with significantly reduced *XTH18* mRNA levels, developed shorter epidermal root cells, possibly due to cell elongation defects (Osato et al., 2006). Interestingly, while both *XTH18* and *19* expression were induced by auxin treatment, only *XTH19* expression was altered in an *axr2-1* auxin gain-of-function mutant (Yokoyama and Nishitani, 2001; Osato et al., 2006). This is in accordance with the presence of AREs in the *XTH19* promoter, while the *XTH18* promoter does not contain such elements. *XTH18* expression is therefore not likely to be directly regulated by auxin. However, GA enhances *XTH18* expression, and since auxin controls root growth, at least in part, via a GA-dependent DELLA protein pathway, this could be one link between the two pathways (Yokoyama and Nishitani, 2001; Fu and Harberd, 2003). These observations suggest that, while the four *XTH*s have evolved via gene duplication, the control of their expression has changed over time, which possibly reflects their involvement in different developmental pathways (Yokoyama and Nishitani, 2001; Vissenberg et al., 2005).

Auxin may also regulate peroxidase-mediated cell wall loosening. Interestingly, class III peroxidases, which are involved in cell wall modification, can mediate both wall loosening and rigidification (Passardi et al., 2004). Auxin is most likely involved in this process by regulating the production of **⋅**O_2_^-^ via NADPH during the oxidative cycle, which the peroxidases can use to generate hydroxyl radicals (**⋅**OH) via H_2_O_2_. The release of these reactive oxygen radicals can result in enzyme-independent cell wall loosening by cleavage of pectins and/or hemicellulose (Chen and Schopfer, 1999). This effect can be counter-acted by increased concentrations of ascorbic acid in the apoplastic space (Joo et al., 2001). Ascorbates were shown to scavenge ROS, thereby inhibiting cell elongation (Joo et al., 2001). On the other hand, the peroxidases can oxidize two tyrosine residues in the extensin glycoproteins of the cell wall, using H_2_O_2_ as an oxidant, to form inter-molecular bridges between the extensins, and thereby rigidifying the cell wall, which has been shown *in vitro*, using isolated peroxidases (Schnabelrauch et al., 1996). In addition, increased apoplastic H_2_O_2_ concentrations correlated with reduced cell elongation (Chen and Schopfer, 1999). At least in onion epidermis cells, this action is again counter-acted by ascorbate, which can inhibit peroxidase activity, thereby indirectly promoting cell elongation (Córdoba-Pedregosa et al., 1996). While the exact effect on root growth remains to be studied, it is plausible that peroxidases generate free radicals in the EZ to loosen the cell walls, while they function in wall stiffening at, or after, the transition from the EZ to the DZ.

## The Root Differentiation Zone

Cells will enter the DZ once they have reached their final size at the end of the EZ, and become functionally specialized. Many *XTH*s are expressed in cells that have reached the DZ, including *XTH14*, *XTH17*, *18*, *19* and *20*, and *XTH26*. Of these, *XTH14*, *XTH20*, and *XTH26* are specifically expressed in the DZ, with *XTH20* expression being restricted to the vascular bundles within this zone (Vissenberg et al., 2005; Becnel et al., 2006). It may be anticipated that cell wall extensibility would be less intense in the DZ as compared to the EZ. The *XTH* expression could therefore either indicate that some cell wall extensibility is still required for certain cells to reach their final fate, or that the XTH activity might be needed for cell wall stabilization. A possible role of XTH14 and XTH26 in this latter process has been demonstrated by adding the active enzymes to growing roots, which led to wall tightening and growth inhibition (Maris et al., 2009). Interestingly, while expression of *XTH26* seems to be auxin-induced, *XTH14* expression is not linked to hormone pathways (Yokoyama and Nishitani, 2001). Apart from the putative XTH activity, cross-links will be introduced into the cell wall to further provide stability. One way of promoting this is via peroxidase-mediated cross-linking of wall extensins that already was discussed above; however, another form of cross-linking is via Ca^2+^-links between pectic HGs. Pectins are typically esterified when they are incorporated into the cell wall (Goldberg et al., 1996). *In muro*, PMEs de-esterify the pectins, allowing them to create Ca^2+^-linkages between the unesterified carboxyl groups and thereby form a gel-like matrix around the cellulose-xyloglucan network that makes the cell wall denser and reduce its viscoelasticity (Smidsrød and Haug, 1971; Goldberg et al., 1986; Catoire et al., 1998). Interestingly, introduction of alkynylated fucose residues into pectic RG-I revealed that cells of the DZ produced these polymers in parallel to cortical microtubules, similar to newly synthesized cellulose microfibrils (Anderson et al., 2012). This would indicate that pectins do not only work as a semi-viscous matrix around the cellulose/xyloglucans, but also surrounds cellulose microfibrils and that cell wall incorporation of the polymers occur with a certain directionality (Anderson et al., 2012).

Another way of generating a stable cell wall is through the deposition of a lignified secondary cell wall (Kumar et al., 2015). Only some specialized cells produce secondary cell walls; in *Arabidopsis* roots, secondary cell walls are produced in the vasculature around the xylem vessel cells that transport water and nutrients to the aerial parts of the plant (Wardrop and Harda, 1965). Like primary cell walls, secondary walls contain cellulose and hemicelluloses; with xylan and mannans being the major hemicelluloses in contrast to xyloglucans in primary cell walls of *Arabidopsis*. However, secondary walls typically contain low levels of pectins and instead hold poly-phenolic lignin polymers (Bidlack et al., 1992). Lignin, being a hydrophobic polymer, can effectively prevent the cells from loosing water and are therefore ideal to seal off the tracheary vessels for efficient water transport. Also, lignin covalently cross-link cell wall polymers, thereby providing the wall with additional strength (Kumar et al., 2015). The NAC-transcription factor VND7 has previously been identified as a major regulator of secondary wall biosynthesis in *Arabidopsis*, binding directly to the promoters of several genes involved in secondary wall formation (Yamaguchi et al., 2011). Indeed, overexpression of *VND7* is sufficient to induce xylem vessel differentiation in root and other plant cells (Yamaguchi et al., 2011). More recently, in a large-scale network-based approach, testing the binding of almost 500 xylem-specific transcription factors against a library of promoter DNA sequences, Taylor-Teeples et al. (2015) revealed a more complex map of transcriptional interactions regulating secondary cell wall formation (Taylor-Teeples et al., 2015). In this network, the E2Fc transcription factor was identified as a master regulator upstream of VND7. Furthermore, E2Fc regulates several lignin, cellulose and hemicellulose biosynthesis genes not only through VND7 (and VND6), but also directly, thereby creating a fast-forward loop as an additional layer of transcriptional regulation; a common theme found within this comprehensive network (Taylor-Teeples et al., 2015). The onset of secondary cell wall production is, at least in part, controlled by different phytohormones, with cytokinins and auxin playing important roles (Jung et al., 2008; Ranocha et al., 2013). For example, floral tissue showed reduced secondary wall thickening when overexpressing AHP4, a positive regulator of cytokinin signaling (Jung et al., 2008). Conversely, *ahp4* mutants displayed enhanced lignification (Jung et al., 2008). Since *AHP4* is also expressed in root tissue, and the *ahp4* plants exhibit defects in xylem vessel development, it may be anticipated that AHP4 also plays a role in secondary wall formation of xylem vessel cells in the root vasculature (Tanaka et al., 2004). Cytokinin signaling is also important in root vasculature development, in particular for xylem specification (Ohashi-Ito and Bergmann, 2007). In xylem precursor cells the transcription factors LHW and T5L1 function in a complex to specify xylem cells and promote cell proliferation in the procambial cells via cytokinin signaling (Ohashi-Ito and Bergmann, 2007; Ohashi-Ito et al., 2014). LHW/T5L1 induces cytokinin biosynthesis genes in the xylem precursors, while at the same time inhibiting cytokinin signaling directly in the xylem precursor cells by promoting expression of *AHP6*, a negative regulator of cytokinin signaling (Ohashi-Ito et al., 2014). Hence, cytokinin functions non cell-autonomously in the procambium cells and possibly in the xylem. Auxin affects secondary wall synthesis, evident for example via the *walls are thin1* (*wat1*) mutant; although it is not clear if the mutant also impacts on root secondary wall formation (Ranocha et al., 2013). WAT1 is a vacuolar auxin transporter and *wat1* mutants displayed reduced secondary wall thickness of xylary fibers in stems, which could be rescued by applying exogenous auxin (Ranocha et al., 2013). The secondary walls in the vasculature of the roots were not explicitly analyzed in this publication, but *WAT1* is strongly expressed in the vasculature of both shoot and root, indicating that it might function in a similar pathway also in the root. BRs may also be regulating wall synthesis in roots by promoting the expression of cell wall synthesis genes via the transcription factor BES1. BES1 acts downstream of BR-signaling and was found in ChIP-chip experiments using whole seedlings to bind to the promoters of several genes involved in cell wall synthesis (Yu et al., 2011). Accordingly, *bes1* mutants have reduced cellulose content in their cell walls.

The DZ is furthermore marked by the emergence of specialized cells and structures, such as lateral root founder cells, the CS and root hairs. The cell wall dynamics in the latter cell type have been extensively covered in other recent reviews (Ishida et al., 2008; Mendrinna and Persson, 2015; Mangano et al., 2016; Salazar-Henao and Schmidt, 2016) and we have therefore chosen not to include root hairs in this review. Lateral roots are initiated in the inner pericycle cell file (Malamy and Benfey, 1997). The emerging root therefore has to break through the cell walls of at least three cell layers; the endodermis, cortex and epidermis. Auxin is involved in this process: first, shoot-derived auxin specifies the lateral root hair founder cells in the pericycle by creating a local auxin maximum (De Smet et al., 2007), second, auxin from these founder cells then primes the overlying endodermal cells for subsequent breakthrough of the emerging lateral root (Swarup et al., 2008). This priming is achieved by auxin-signaling from the pericycle to the endodermis via the auxin importer LAX3 and activation of the SHY2/IAA3 pathway, which then results in a loss in volume of the endodermis cells, in turn accommodating swelling and subsequent outgrowth of the lateral root (Swarup et al., 2008; Vermeer et al., 2014). Another part of priming the overlying cell layers for the outgrowth of the lateral root is the transcriptional regulation of several cell wall modifying proteins. These include EXPANSINs, pectate lysases, and, indirectly affecting cell wall integrity, aquaporins (Swarup et al., 2008; Péret et al., 2012). Up-regulation of *EXPANSIN* expression results in local cell wall loosening at the position where new lateral roots will emerge. Additionally, transcriptional repression of aquaporins leads to reduced water transport between the primordium and the cells above it, thereby changing the cells turgor, resulting in reduced cell wall stability (Péret et al., 2012). Finally, the local expression of pectate lyases in the cells overlying the emerging new lateral root results in wall destabilization by modifying pectins. The importance of de-esterified pectins in stabilizing the cell wall via calcium cross-links was already discussed above. However, there is another, contrary effect of pectin de-esterification: de-esterification of cell wall pectins can also result in local eliminative cleavage of pectins by the activity of pectate lyases, and therefore in the removal of the middle lamella between two cells (Domingo et al., 1998). Both effects are important for lateral root development (Laskowski et al., 2006). Cross-linking of the de-esterified pectins in the cells of the primordium results in a more stable cell wall, enabling the young root to break through the overlying tissue. At the same time, local expression of pectate lyases in the cells overlying the emerging root results in the local degradation of the cells middle lamella, allowing the lateral root to break through between these cells (Laskowski et al., 2006).

An interesting feature of the endodermal cells in the DZ is the appearance of the CSs (Caspary, 1865). The CSs are modified primary anticlinal cell walls of the endodermis. The strips therefore connect the single endodermal cells with an impenetrable barrier, which seal off the apoplastic space otherwise connecting the central cylinder with the outer parts of the root and the environment (de Rufz de Lavison, 1910; Nagahashi and Thomson, 1974). Formation of the CS is dependent on the so-called CASPs, which accumulate at the plasma membrane in a region adjacent to the cell wall domain in which the CS will be formed (Roppolo et al., 2011). The CASPs directly interact with each other and remain tightly linked to the cell wall, as they do diffuse laterally in the membrane. Most likely they function to guide components of the lignin biosynthesis machinery to the CS domain, where they deposit monolignols into the wall (Roppolo et al., 2011). Furthermore, NADPH oxidases and peroxidases are involved in CS formation. The NADPH oxidases which localize to the CS domain, potentially also dependent on CASP function, generate ROS to produce H_2_O_2_, which in turn serves as substrate for the peroxidases. The peroxidases then aid the polymerization of the monolignols to form an impermeable lignin impregnation between two cell walls (Lee et al., 2013). Interestingly, endodermis cell walls go through a second level of differentiation by deposition of hydrophobic polymer suberin around the surface of endodermal cells (Andersen et al., 2015). Suberin is deposited as secondary cell walls in the form of lamellae in between primary cell wall and plasma membrane. In contrast to the CSs, which blocks the apoplastic diffusion of ions through endodermis, suberin lamellae resist ion uptake into the endodermal cells (Barberon et al., 2016). This implies that once the endodermal diffusion barriers are fully formed, the endodermis will not be permeable for ions and active uptake will be needed.

## Cell Walls and Nutrient Transport

A major function of the plant roots is to absorb water and essential nutrients from the soil. Nutrients are absorbed at the soil-root interface and radially transported toward the central stele. The radial transport occur through three pathways; (i) An apoplastic pathway where nutrients passively diffuse through the apoplast (i.e., through the cell walls); (ii) A symplastic pathway that involves selective uptake of nutrients by cells through transporters (once inside the cells, molecules and ions may move from cell to cell toward the central stele through the PD); (iii) A coupled *trans*-cellular pathway that involves repeated import and export of nutrients from the cells through the action of influx and eﬄux carriers (Barberon and Geldner, 2014).

Soils typically contain a mixture of nutrients, but also unwanted compounds and pathogens. It is, therefore, essential for roots not only to selectively take up the required nutrients but also to prevent them from releasing them back into the soil. As indicated above, endodermal cell wall differentiation transform roots into selectively absorbing organs (Barberon and Geldner, 2014; Andersen et al., 2015). Here, the CSs effectively block the apoplastic transport of ions into the stele (Alassimone et al., 2010; Naseer et al., 2012). Hence, water and nutrients need to be transported across the endodermis through a more selective, symplastic pathway. *Schengen3 (sgn3)* mutants that have discontinuous CSs display potassium (K) deficiency in the leaves, also when grown on soils with high potassium levels. The study hypothesized that without the presence of intact CSs, K is continuously leaking back into the soil. This demonstrates the role of the CSs in maintaining nutrient homeostasis inside the plants (Pfister et al., 2014). Except for the mild K starvation, surprisingly, most nutrients were maintained at wild-type level in the *sgn3* mutants and plants showed normal growth and seed set (Pfister et al., 2014). This suggests that plants are able to compensate for defects in the CSs, at least under ideal growth conditions. However, how these compensation mechanisms are regulated remains to be investigated.

Recently, Barberon et al. (2016) reported that the deposition of endodermal suberin can be altered in response to the nutritional status of plants, and this plasticity is antagonistically regulated by ABA and ethylene signaling. Using mutants affected in nutrient transport and hormone signaling, the study showed that potassium (K) and sulphur (S) deficiencies lead to increased suberization through ABA-mediated signaling, whereas iron (Fe), zinc (Zn), or manganese (Mn) deficiency inhibits suberization through ethylene signaling. Furthermore, the growth of Fe transport mutants was rescued in plants that specifically degrade suberin in the endodermis (Barberon et al., 2016). This indicates that reduced suberization might be beneficial for plants grown under Fe deficient condition. In addition, suberization of the endodermis is also important for defense against pathogen infection. Here, suberization increases upon infection of roots by certain pathogens, and plants with increased endodermal suberization have increased resistance to these pathogens (Ranathunge et al., 2008). Furthermore, endodermis suberization is also important for controlled colonization of the root by the *Arabidopsis* symbiotic fungus *Ct.* Most of the *Ct* hyphae were unable to penetrate suberized cell walls of the endodermis (Hiruma et al., 2016). Nevertheless, some hyphae were able to cross the endodermis and colonize the stele, probably through occasional unsuberized endodermal cells, called passage cells (Andersen et al., 2015). These findings demonstrate that endodermal suberization is physiologically significant and an extraordinary ability of plants to adopt suberization in response to environmental constraints.

During nutrient starvation, plants are able to adapt their root system to explore soil zones for nutrients. These nutrient concentration-dependent changes in the root architecture and morphology are to some extent nutrient specific. For example, phosphate (Pi) deficiency leads to stunted primary root growth, and an increase in lateral roots and root hairs, while nitrate starvation leads to longer lateral roots (Gruber et al., 2013). Here, modifications of the cell walls are required for the growth adaptation of the roots to occur. This notion is further supported by a substantial number of changes in the expression of cell wall-related genes in roots of nutrient signaling mutants, and in response to changes in nutrient availability (Trevisan et al., 2015; Salazar-Henao and Schmidt, 2016; Wege et al., 2016). Apart from transcriptomic analyses, very little work has been done to understand cell wall responses to nutrients, and mechanisms behind such responses are therefore lacking.

Phosphate starvation is one of the best characterized areas related to nutrients and cell walls. Here, plants substantially adjust their root architecture and also modulate the cellulose content (Zhang et al., 2012). Furthermore, mutants affected in cellulose synthesis showed a constantly active Pi starvation response and increased Pi transport, even when grown on high levels of Pi (Jin et al., 2015). This suggests that the Pi starvation-signaling pathway is, at least in part, linked to the cell wall integrity. This hypothesis is corroborated by the observation that *Arabidopsis* plants grown under Pi-deficient conditions showed increased thickening of primary cell walls and callose deposition in the walls around the PD (Müller et al., 2015). Mutants with altered root growth response to Pi starvation also showed altered cell wall organization. PDR2 encodes a putative P5 ATPase, and mutations in this gene displayed hypersensitive responses to low Pi, including stunted root growth, and increased cell wall thickening and callose deposition in the walls surrounding PD (Ticconi et al., 2004; Müller et al., 2015). In contrast, primary root growth was not arrested in *low phosphate root 1 (lpr1)* mutants, and they did not show any increase in callose deposition or in cell wall thickness that was observed in wild-type plants (Müller et al., 2015). LPR1 encodes for a ferroxidase that might initiate Fe redox cycling as a potential source of ROS during Pi starvation. ROS production might be therefore be needed for the initiation of callose deposition in the cell wall leading to reduced transport through PD and meristem activity (Müller et al., 2015). However, the exact basis of Pi starvation-mediated cell wall thickening and the signaling components involved in this process remain elusive. Furthermore, how Pi is perceived and its impact on LPR1 and PDR2 remains to be investigated.

## Conclusion and Outlook

Cell walls are a defining feature of all plants and constitute the bulk of a plant’s biomass. While the general components of the cell wall are largely known, it is clear that the walls of different cell types differ significantly, depending on tissue, developmental state and environment. Surprisingly, relatively little is known about cell wall re-modeling and contents of roots, even in the model plant species *Arabidopsis*. Consequently, the underpinning pathways and mechanisms that drive root cell wall plasticity are not well-known. Moreover, root cell walls have a remarkable ability to adapt to environmental constraints and nutrient fluctuations. These adaptations are clearly of physiological importance, and may have great implications for agriculture as root architecture drives plant growth. While some work has linked action of different phytohormones to cell wall changes (as reviewed in Sánchez-Rodríguez et al., 2010), direct effects are largely missing. Hence, efforts to better understand cell wall synthesis, re-modeling and the pathways that drive these processes may result in tailoring plant root growth and therefore plant performance.

## Author Contributions

SP and MS conceived the topic of the review. GK and MS wrote the manuscript together with SP.

## Conflict of Interest Statement

The authors declare that the research was conducted in the absence of any commercial or financial relationships that could be construed as a potential conflict of interest.

## ABBREVIATIONS

ABA: abscisic acid
AGP: arabinogalactan-protein
AHP4: ARABIDOPSIS HISTIDINE-CONTAINING PHOSPHOTRANSFER FACTOR 4
ARE: auxin response element
*AXR2*: *AUXIN RESISTANT 2*
*Bot1*: *botero1*
BES1: BRI1-EMS-SUPPRESSOR1
BR: brassinosteroid
*Bul*: *boule*
Ca^2+^: calcium ion
CASP: CASPARIAN STRIP DOMAIN PROTEIN
CesA: cellulose synthase
ChIP: chromatin immunoprecipitation
COB: COBRA
CS: casparian strip
CSI1: Cellulose Synthase Interacting1
*Ct*: *Colletotrichum tofieldiae*
DZ: differentiation zone
EZ: elongation zone
GA: gibberellic acid
H_2_O_2_: hydrogen peroxide
HG: homogalacturonan
IAA7: INDOLE-3-ACETIC ACID 7
LAX3: LIKE AUX1 3
LHW: LONESOME HIGHWAY
LPR1: LOW PHOSPHATE ROOT 1
MZ: meristematic zone
NADPH: nicotinamide adenine dinucleotide phosphate
**⋅**O_2_^-^: superoxide
**⋅**OH: hydroxide
PD: plasmodesmata
PDR2: PHOSPHATE DEFICIENCY RESPONSE 2
PMEs: pectin methyl esterases
*Prc1*: *procuste1* (CesA6)
QC: quiescent center
RAM: root apical meristem
RGI: rhamnogalacturonan I
RGII: rhamnogalacturonan II
ROS: reactive oxygen species
SGN3: SCHENGEN3
SHR: SHORT-ROOT
SHY2: SHORTHYPOCOTYL 2
T5L1: TARGET OF MONOPTEROS5-LIKE1
TZ: transition zone
VND7: VASCULAR-RELATED NAC-DOMAIN7
*WAT1*: *WALLS ARE THIN 1*
XEH: xyloglucan endohydrolase
XET: xyloglucan endotransglucosylase
XTH: Xyloglucan endotransglucosylase/hydrolase.
